# Multi-Input Distributed Classifiers for Synthetic Genetic Circuits

**DOI:** 10.1371/journal.pone.0125144

**Published:** 2015-05-06

**Authors:** Oleg Kanakov, Roman Kotelnikov, Ahmed Alsaedi, Lev Tsimring, Ramón Huerta, Alexey Zaikin, Mikhail Ivanchenko

**Affiliations:** 1 Oscillation Theory Department, Lobachevsky State University of Nizhniy Novgorod, Nizhniy Novgorod, Russia; 2 Department of Bioinformatics, Lobachevsky State University of Nizhniy Novgorod, Nizhniy Novgorod, Russia; 3 Department of Mathematics, King AbdulAziz University, Jeddah, Saudi Arabia; 4 BioCircuits Institute, University of California San Diego, La Jolla CA, USA; 5 Institute for Women’s Health and Department of Mathematics, University College London, London, United Kingdom; Imperial College London, UNITED KINGDOM

## Abstract

For practical construction of complex synthetic genetic networks able to perform elaborate functions it is important to have a pool of relatively simple modules with different functionality which can be compounded together. To complement engineering of very different existing synthetic genetic devices such as switches, oscillators or logical gates, we propose and develop here a design of synthetic multi-input classifier based on a recently introduced distributed classifier concept. A heterogeneous population of cells acts as a single classifier, whose output is obtained by summarizing the outputs of individual cells. The learning ability is achieved by pruning the population, instead of tuning parameters of an individual cell. The present paper is focused on evaluating two possible schemes of multi-input gene classifier circuits. We demonstrate their suitability for implementing a multi-input distributed classifier capable of separating data which are inseparable for single-input classifiers, and characterize performance of the classifiers by analytical and numerical results. The simpler scheme implements a linear classifier in a single cell and is targeted at separable classification problems with simple class borders. A hard learning strategy is used to train a distributed classifier by removing from the population any cell answering incorrectly to at least one training example. The other scheme implements a circuit with a bell-shaped response in a single cell to allow potentially arbitrary shape of the classification border in the input space of a distributed classifier. Inseparable classification problems are addressed using soft learning strategy, characterized by probabilistic decision to keep or discard a cell at each training iteration. We expect that our classifier design contributes to the development of robust and predictable synthetic biosensors, which have the potential to affect applications in a lot of fields, including that of medicine and industry.

## Introduction

The current challenge facing the synthetic biology community is the construction of relatively simple, robust and reliable genetic networks, which will mount a pool of modules, potentially to be connected into more complex systems. Rapid progress of experimental synthetic biology has indeed provided several synthetic genetic networks with different functionality. Since the development of two fundamental simple networks, representing the toggle switch [1] and the repressilator [2] in 2000, a vast number of proof-of-principle synthetic networks have been designed and engineered. Among them transcriptional or metabolic oscillators [3–5], spatially coupled and synchronised oscillators [6, 7], calculators [8], inducers of pattern formation [9], learning systems [10], optogenetic devices [11], memory circuits and logic gates [12–15].

One of the much awaited kinds of synthetic gene circuits with principally new functionality would work as intelligent biosensors, for example, realized as genetic classifiers able to assign inputs with different classes of outputs. Importantly, they would need to allow an arbitrary shape of the area in the space of inputs, in contrast to simple threshold devices. Recently, the first step in this direction has been made in [16], where the concept of a distributed genetic classifier formed by a heterogeneous population of genetically engineered cells has been proposed. Each cell in the distributed classifier is essentially an individual binary classifier with specific parameters, which are randomly varied among the cells in the population. The inputs to the classifier are certain chemical concentrations, which the engineered cells can be made sensitive to. The classification output from an individual cell can be provided, for example, by the fluorescent protein technique which is well developed and universally adopted in synthetic biology. The output of the whole distributed classifier is the sum of the individual classifier outputs, and the overall decision is made by comparing this output to a preset threshold value. If the initial (or “master”) population contains a sufficiently diverse variety of cells with different parameters, the whole ensemble can be trained by examples to solve a specific classification problem just by eliminating the cells which answer incorrectly to the examples from the training sequence.

Note that strictly speaking, the selection procedure does not realize any kind of learning at the level of individual classifier (cell). On the other hand, we view the whole ensemble as a distributed classifier, and reshaping population can be regarded as tuning its parameters. Since reshaping occurs in response to a sequence of training examples, we refer to this procedure as learning.

The paper [16] focused on distributed classifiers composed of single-input elementary classifiers. The single-input genetic circuit proposed in [16] provides a bell-shaped output function against the input chemical concentration. The individual cells in the population differ from each other by the choice of the particular input chemicals that they are sensitive to, and by the width and positioning of the bell-shaped response function. These parameters can be varied in a range of up to 10^5^ by modifying the ribosome binding sites in the gene circuit [17, 18]. Such libraries of cells with randomized individual parameters have been constructed in experiments for synthetic circuit optimization [19–21]. The single-input distributed classifier has been numerically tested on several examples in [16].

However, practical applications may require classification of multiple inputs. In [16] it has been discussed that the same principles can be utilized for a design of two- or multi-input circuits. The proposed circuit is based upon a genetic AND gate [22–24], providing a bell-shaped response function in the space of two or more inputs. Nevertheless, no studies of a distributed classifier with two or more inputs have been performed so far. In this paper we fill this gap by developing distributed classifiers based upon two types of elementary two-input classifier cells: one is a simple scheme implementing a linear classifier in the space of two inputs and the other is the scheme with AND gate and bell-shaped response proposed in [16].

Below we consider two settings of the classification problem. In the first setting, which we refer to as “hard classification”, the classes are assumed separable, which implies that the sets of points in the parameter space belonging to either class do not intersect. In this case all elementary classifiers can be unambiguously separated into those answering correctly and incorrectly to the training examples, and the “hard learning strategy”, which is based upon discarding all incorrectly answering cells, may be used.

We start with considering the case of separable classes and hard learning, using linear classifiers as elementary cells. We show that using this strategy a range of separable classification problems can be reliably solved even with a small number of elementary classifiers, including problems which become inseparable (and, thus, imposing a lower bound on the error rate) when attempted to be solved by single-input classifiers. At the same time, this approach is incapable of solving classification problems with more complicated classification borders, as well as problems with inseparable classes.

In the second part we address both mentioned issues by means of soft learning strategy and elementary cells with bell-shaped response. We demonstrate the effectiveness of this approach for solving these more complicated tasks at the expense of a more complicated gene circuit in each elementary classifier and a greater number of cells required.

## Hard classification problem

### Two-input linear classifier circuit

**Fig 1:**
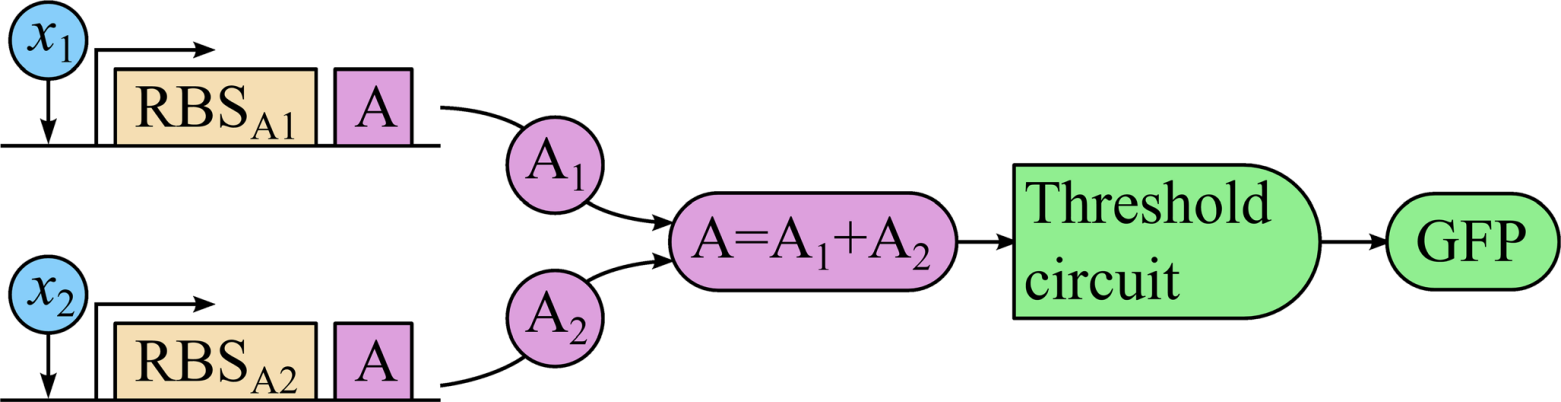
Scheme of a two-input linear classifier circuit. *x*_1_, *x*_2_—inputs inducing the corresponding promoters, RBS_A1_ and RBS_A2_—ribosome binding sites determining the strengths of the input branches, A—intermediate transcription factor (same in both input branches), GFP—reporter gene.

We assume that the classifier input is a set of chemical concentrations capable of regulating appropriate synthetic promoters (directly or mediated by the regulatory network of the cell). In the simplest design of a multi-input genetic classifier circuit, the input genes drive the synthesis of the same intermediate transcription factor *A* (Fig 1), but are regulated by different promoters sensitive to the corresponding input chemicals *X*_*j*_. The expression of the reporter protein, for example, green fluorescent protein (GFP), is driven by the total concentration of *A*, summarized from all input genes.

The stationary concentration *a* of the intermediate transcription factor can be expressed as a weighted sum over all classifier inputs

$$\begin{array}{c} a=\sum_{j}b_{j}a_{j}\left(x_{j}\right), \end{array}$$ (1)

where *x*_*j*_ are concentrations of the inputs *X*_*j*_, *a*_*j*_(⋅) are nonlinear functions, each describing the response to a particular input, including the whole appropriate signalling pathway, and *b*_*j*_ are linear multipliers determining the relative strengths of the corresponding inputs, which can be varied in a range of more than 10^5^-fold by varying the DNA sequence within and near the ribosome binding site of the corresponding input gene [17, 18].

For a sharper discrimination between the classifier decisions, we propose to make use of the protein sequestration technique [25] to generate an ultrasensitive response to *A* when its concentration exceeds a certain threshold. This is achieved by binding *A*, which normally induces the reporter gene, with a suitable inhibitor into an inactive complex which can not bind DNA. The simplest description of this binding assumes that free active transcription factor *A* becomes available only when all inhibitor molecules are bound. Then the reporter protein concentration *g* may be approximated by a shifted and truncated Hill function [25]

$$\begin{array}{c} g=g\left(a;\theta \right)=\{\begin{array}{cc} \alpha \gamma , & \text{if}\;a\leq \theta ,\\ \gamma \frac{\alpha A_{g}+a-\theta }{A_{g}+a-\theta }, & \text{if}\;a>\theta , \end{array} \end{array}$$ (2)

where *θ* is the threshold determined by the constitutive expression rate of the inhibitor [25], *A*_*g*_ is the DNA-binding dissociation constant for *A*, *γ* determines the maximal output, and *αγ* is the basal expression of the reporter protein in the absence of *A*.

Response function of type Eq (2) was derived and experimentally tested in [25] using a dimeric transcription factor CEBP*α* along with a specially designed inhibitor, both from the basic leucine zipper protein family. The applicability of Eq (2) is conditioned by a specific hierarchy of dissociation constants and typical concentrations of proteins [25]. Namely, (i) sequestration being the dominant reaction (dissociation constant significantly smaller than all other relevant dissociation constants and concentrations), so that no free transcription factor is available unless all amount of inhibitor is bound; (ii) dimerization of inhibitor being negligible (dissociation constant significantly greater than all other relevant scales); (iii) dimerization of CEBP*α* being at an intermediate level of affinity (dissociation constant significantly less than typical concentrations of CEBP*α* and inhibitor, all of them at the same time falling in the range set forth by requirements (i) and (ii) above), so that almost all above-threshold amount of CEBP*α* comes in dimerized (active) form. Note, that cooperative activation by dimeric CEBP*α* was deliberately suppressed in [25] by using a promoter with a single binding site. That said, under the above listed conditions the output Eq (2) is expected to retain its form (not exhibiting a Hill index greater than one) even when using a two-site promoter which binds CEBP*α* dimer in a cooperative way, see Supplementary Information in [25]. In other conditions a Hill index greater than one may have to be introduced in Eq (2), in this case the ultrasensitive response sharpens even further.

A master population of cells with randomized individual response characteristics can be obtained by randomly varying the input weights *b*_*j*_, as well as the threshold *θ*, among the cells in the population. In the following we restrict ourselves to the case of two inputs, but our approach equally applies to input vectors of any dimension. We assume that the parameter values in the *i*th individual cell are $b_{1}^{i}$ and $b_{2}^{i}$ for the input weights and *θ* ^*i*^ for the threshold, the lower index denoting the input and the upper one labeling the cells, all other parameters being the same in both input channels in all cells. The GFP output of a chosen *i*th individual classifier cell is then

$$\begin{array}{c} f_{i}\left(x_{1},x_{2}\right)=g\left(b_{1}^{i}a_{1}\left(x_{1}\right)+b_{2}^{i}a_{2}\left(x_{2}\right);\theta^{i}\right) \end{array}$$ (3)

with *g*(*a*;*θ*) defined in Eq (2).

We use the discrete-output model of the individual cell to analyze the learning process and the distributed classifier behaviour. Namely, we assume that each individual cell can produce two distinguishable kinds of output, corresponding to the cases in Eq (2): low, or “negative answer” (which is the subthreshold background output *g*_*i*_ = *αγ*), and high, or “positive answer” (above-threshold output).

We note that each individual cell acts as a linear classifier in the transformed input space with coordinates (*a*_1_,*a*_2_) defined by the corresponding nonlinear input functions

$$\begin{array}{c} a_{1}=a_{1}\left(x_{1}\right),\;a_{2}=a_{2}\left(x_{2}\right). \end{array}$$ (4)

Indeed, an individual *i*th cell generates high output when $b_{1}^{i}a_{1}+b_{2}^{i}a_{2}>\theta^{i}$, or

$$\begin{array}{c} m_{1}^{i}a_{1}+m_{2}^{i}a_{2}>1, \end{array}$$ (5)

where $m_{1,2}^{i}=b_{1,2}^{i}/\theta^{i}$.

Such classifier divides the transformed input space into two regions, corresponding to either answer of the classifier, which we will refer to as the negative and the positive classes. The border separating the classes in the transformed input space is a straight line

$$\begin{array}{c} m_{1}^{i}a_{1}+m_{2}^{i}a_{2}=1. \end{array}$$ (6)

Note that *a*_1,2_ as well as *m*_1,2_ can not be negative due to their meaning. In the following, *a*_1,2_ and *m*_1,2_ are assumed to be non-negative real numbers. In particular, it means that the space of inputs and the space of parameters are always limited to the first quadrant (or hyperoctant with all coordinates non-negative) of the full real space, regardless of its dimension.

### Hard classification technique and learning strategy

An ensemble of linear classifiers can be utilized to perform a more complicated classification task with a piecewise-linear border in the transformed input space. Denote with *P*_*i*_ the positive class of the *i*th individual classifier:

$$\begin{array}{c} P_{i}=\{a_{1},a_{2}:m_{1}^{i}a_{1}+m_{2}^{i}a_{2}>1\}. \end{array}$$ (7)

**Fig 2:**
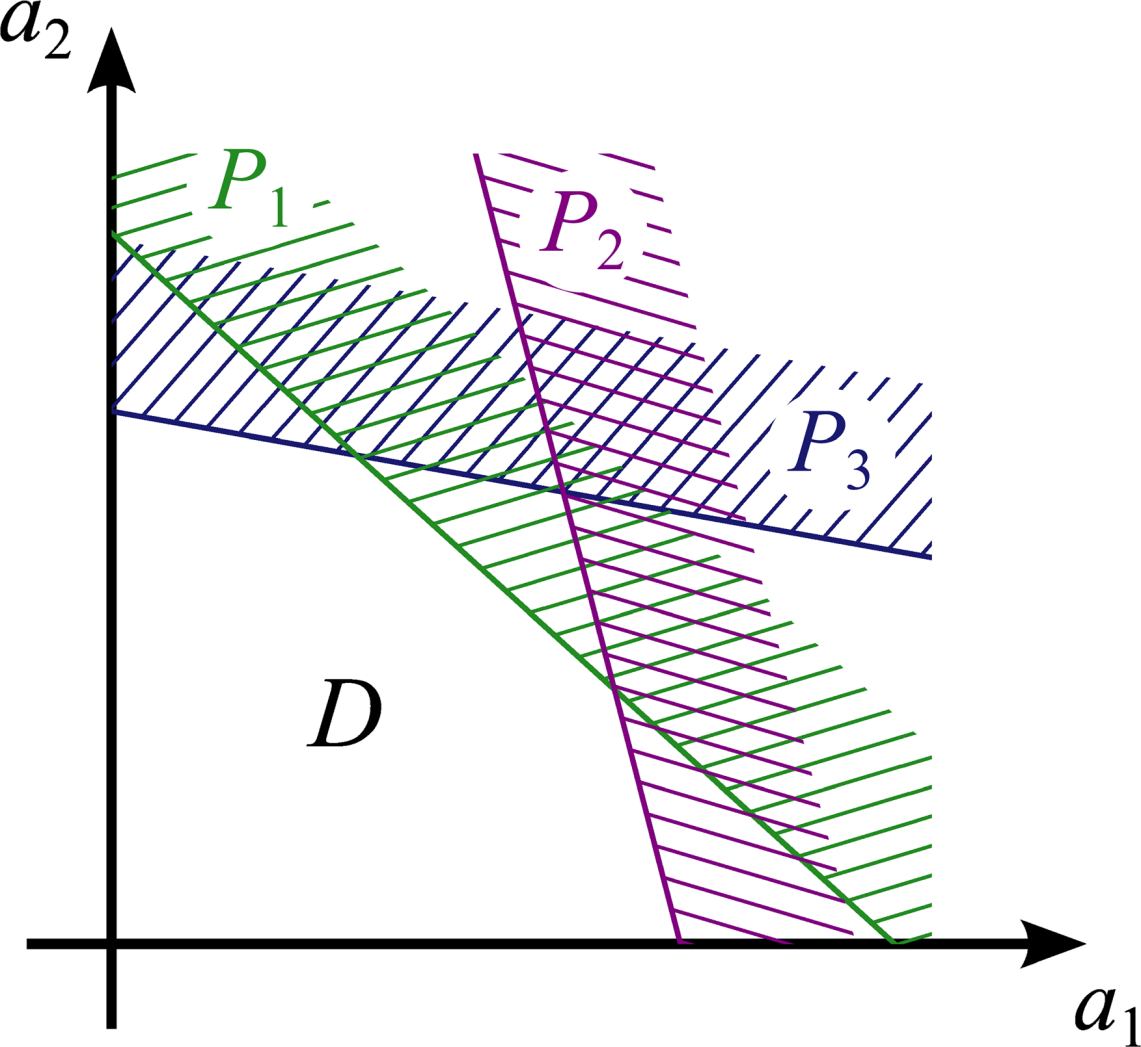
Hard classification technique. *P*_1_, *P*_2_, *P*_3_—positive classes of individual linear classifiers, *D*—negative class of the collective classifier.

Let all elements in the ensemble be given the same input. Then the whole ensemble can be used as a single distributed classifier, dividing the transformed input space into the positive class *P* = ⋃_*i*_ *P*_*i*_, where at least one individual classifier gives the positive answer, and the negative class $D=\bar{P}=\cap_{i}{\bar{P}}_{i}$, where all classifiers answer negatively (here the overbar “$\bar{\;}$” denotes complement in the transformed input space), see Fig 2.

By construction, the negative class *D* is entirely contained in each closed half-plane defined by any of its edges, which means it is always convex. The classification border is a polygonal line composed of segments, each described by an equation of type Eq (6), all having negative slope, because both $m_{1}^{i}$ and $m_{2}^{i}$ are positive. In the limit of large number of cells, the negative class becomes a convex region bordered by the coordinate axes and a smooth classification border having negative tangent slope at each point.

An ensemble constituting a distributed classifier with a specified (“target”) classification border (satisfying the requirements of negative slopes and convexity) can be prepared by the following learning algorithm. Let us start with a master population of linear classifiers of type Eq (7) with random parameters $m_{1}^{i}$, $m_{2}^{i}$ distributed continuously over some interval. The aim of the learning is to keep all individual classifiers which answer correctly to *all* training examples and remove all incorrectly answering ones. To achieve this, we test the whole ensemble against a training sequence of samples from the negative class. All elements which answer positively to at least one negative sample are considered “incorrect” and are removed from the ensemble. This can be done, for example, using the fluorescence-activated cell sorting (FACS) technique. Positive class samples are not needed for learning, since hard classification fundamentally assumes separability of classes.

Actually, it is enough to use only samples located along the classification border. Although training sequences of this kind might be not available in real situations, theoretically, excluding the interior of the negative region from the training sequence leads to achieving the same learning outcome with a smaller number of samples.

The ensemble which remains after this learning procedure forms a distributed classifier with the class border determined by the training sequence. The actual set of cells constituting the trained distributed classifier is essentially the outcome of clipping the master population in the parameter space (*m*_1_,*m*_2_) with a certain mask, which completely characterizes the action of the learning algorithm. In other words, the trained ensemble is a set intersection of the master population with a region in the parameter space, which we will refer to as the “trained ensemble region”.

**Fig 3:**
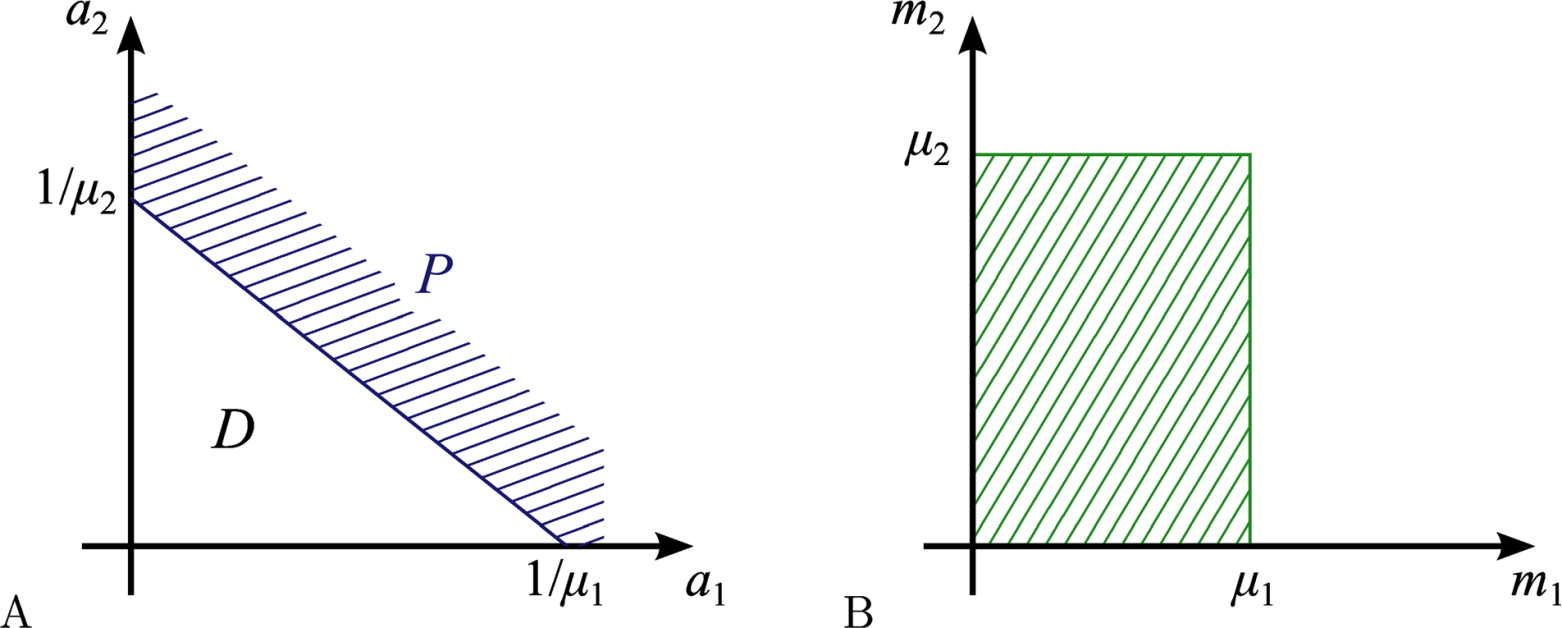
Training a distributed classifier with a linear target border. (A) Target classes: *P*—positive, *D*—negative. (B) Trained ensemble region on the plane of parameters: hatched area.

To get an insight into a quantitative description of hard learning strategy, we start with a trivial case when the target classification border is linear, defined by the equation

$$\begin{array}{c} \mu_{1}a_{1}+\mu_{2}a_{2}=1, \end{array}$$ (8)

where *μ*_1,2_ are given constant coefficients, see Fig 3A. Although this classification task can be solved by a single linear classifier, we use it as a starting point to describe the training of a distributed classifier.

In the course of learning with a sequence of points distributed along the border Eq (8), any element having *m*_1_ > *μ*_1_ or *m*_2_ > *μ*_2_ will eventually answer positively and therefore will be removed from the ensemble. Thus, the trained ensemble region on the plane (*m*_1_,*m*_2_) is a rectangle (hatched area in Fig 3B).

Similarly, if the target border is a polygonal line (satisfying the requirements of negative slopes and convexity), with the target positive class being a union of several linear classes

$$\begin{array}{c} P=\underset{i}{⋃}\{a_{1},a_{2}:\mu_{1}^{i}a_{1}+\mu_{2}^{i}a_{2}>1\}, \end{array}$$ (9)

where $\mu_{1}^{i}$, $\mu_{2}^{i}$ are the coefficients of the individual segments of the target polygonal border, then the trained ensemble region on the plane (*m*_1_,*m*_2_) is a convex polygon with vertices $\left(\mu_{1}^{i},\mu_{2}^{i}\right)$, shown in Fig. S2A in S1 Appendix as hatched area.

In S1 Appendix we analyze the response of a trained hard classifier to an input taken from the positive class. In particular, a lower estimate is obtained for the quantity of cells answering positively to such inputs. It is found to be proportional to the density of the master population per unit of the logarithmic parameter space (log*m*_1_, log*m*_2_). It is also shown that the maximal quantity *m*_max_, to which the region covered by the master population in the parameter space extends in both *m*_1_ and *m*_2_, should be not less than the inverse of the smaller intercept of the target class border (the intercepts are the abscissa and the ordinate of the points where the border crosses the axes *Oa*_1_ and *Oa*_2_).

### Simulations

**Fig 4:**
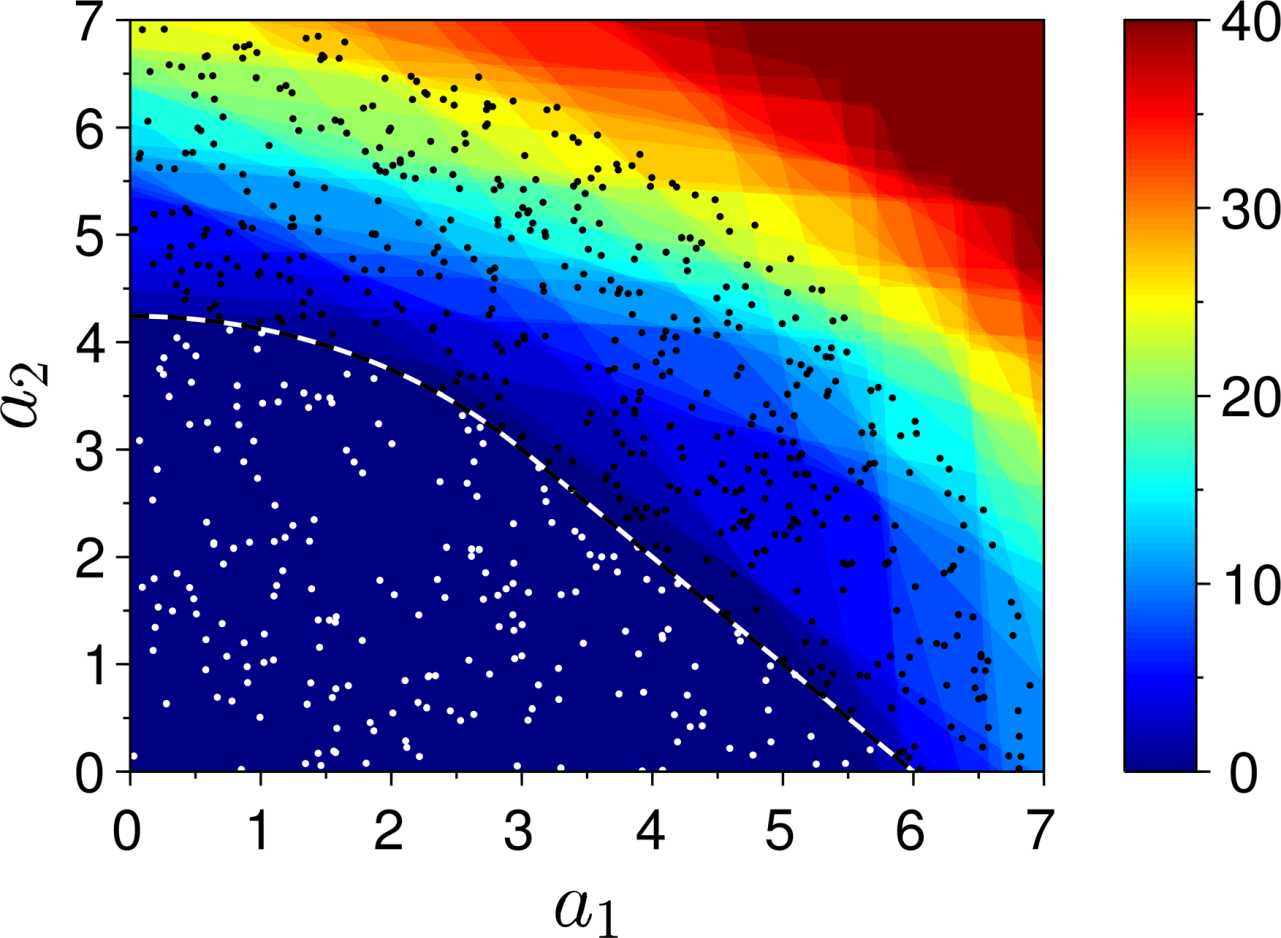
Simulation results for hard classification. Response of a trained distributed classifier in the space of inputs. Black-white dashed line—target (predefined) class border, white (black) filled circles—samples from the negative (positive) class, color—number of the positively responding cells (quantities 40 and above marked with same color).

To illustrate and verify the analytical results, we performed numerical simulations. We specify the class border (black-white dashed line in Fig 4) composed of two sections. One section is a segment of the line *a*_1_+*a*_2_ = *A*, and the other one is an arc of the circle $a_{1}^{2}+a_{2}^{2}=A^{2}/2$. The segments are connected at the point *a*_1_ = *a*_2_ = *A*/2, forming a smooth curve.

The negative class is the bounded part of the first quadrant of the plane (*a*_1_,*a*_2_), separated by the border. The training sequence of length *N*_train_ (white filled circles in Fig 4) is randomly sampled from the negative class. The positive class is additionally bounded by condition $a_{1}^{2}+a_{2}^{2}<B^{2}$ with *B* > *A*.

The master population of the classifier cells is obtained by randomly sampling the parameters (*m*_1_,*m*_2_) from the log-uniform distribution in the parameter space, bounded by the minimal and maximal values *m*_min_ and *m*_max_. The total number of cells in the master population is *N*_master_. The uniform density of cells per logarithmic unit of the parameter space is

$$\begin{array}{c} \alpha =\frac{N_{\text{master}}}{{\left(\log m_{\max}-\log m_{\min}\right)}^{2}}. \end{array}$$ (10)

The classifier is trained by presenting sequentially all training samples from the negative class, and discarding all cells answering positively to at least one sample. Algorithm description in Table 1 formalizes the above procedure.

**Table 1:** Hard learning algorithm.

**Input:** Master population of *N*_master_ elementary linear classifiers (cells) with parameters $\left(m_{1}^{i},m_{2}^{i}\right)$ randomly sampled from the log-uniform distribution in the parameter space, bounded by the minimal and maximal values *m*_min_ and *m*_max_. The training sequence of negative class samples $\left(a_{1}^{j},a_{2}^{j}\right)$ of length *N*_train_.
**Output:** Trained set of cells constituting a distributed classifier.
**for** each training sample $\left(a_{1}^{j},a_{2}^{j}\right)$ **do**
**for** each cell *i* = 1 to *N*_master_ **do**
**if** Eq (5) holds for this cell and this input (cell generates a positive answer) **then**
Remove the cell from the ensemble.
**end if**
**end for**
**end for**

In our simulation we let *N*_master_ = 300, *N*_train_ = 200, *A* = 6, *B* = 8. The smaller border intercept is $A/\sqrt{2}\approx 4.24$. In accordance to the criterion formulated in the end of the previous subsection, we let *m*_max_ = 0.5 > 1/4.24, and *m*_min_ = *m*_max_/100. We measure the quantity of the positively responding cells of the trained classifier as a function of the input (*a*_1_,*a*_2_). The result is depicted in Fig 4 in color code. The straight interfaces of color, distinguishable in the figure, are the borders of type Eq (6) associated with the individual linear classifiers (cells).

## Soft classification problem

The approach considered above can only be applied to hard classification problems with a special type of the classification border (namely, the border must be a curve connecting the axes in the input space, having a negative slope at each point, with the negative class being a convex region, see subsection “Hard classification technique and learning strategy” for details). In order to address problems with classification border of more general type, or “soft” classification problems (i.e. problems with inseparable classes with *a priori* unknown probability distributions in the input space) we employ soft learning strategy and a two-input elementary classifier design with a bell-shaped response function, which was suggested in [16].

### Two-input classifier with a bell-shaped response

**Fig 5:**
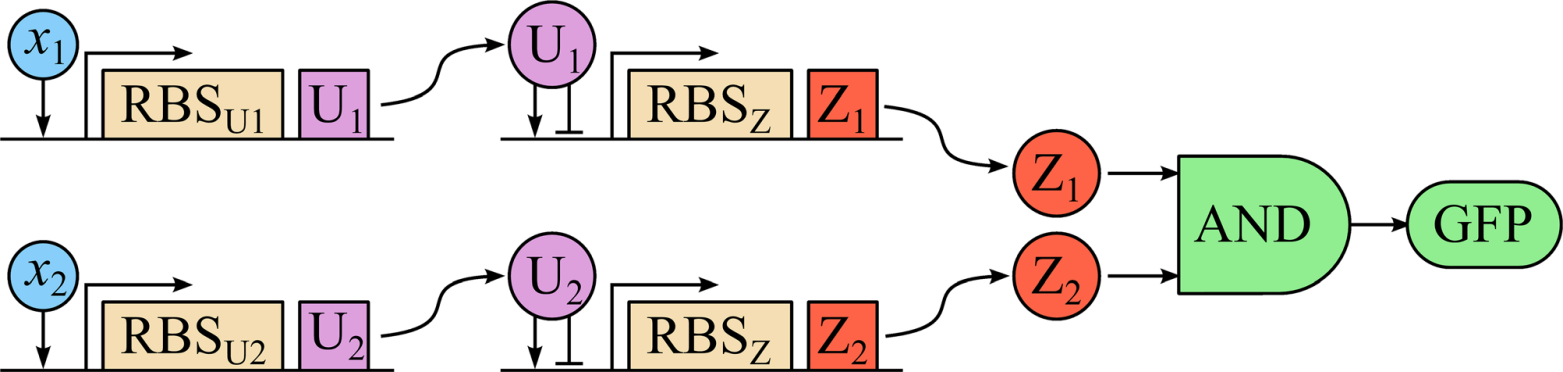
Scheme of a two-input classifier circuit with a bell-shaped response. *x*_1_, *x*_2_—inputs inducing the corresponding promoters, RBS_U1_ and RBS_U2_—ribosome binding sites determining the strengths of the input branches, U_1_, U_2_—intermediate repressor/activator factors, Z_1_, Z_2_—outputs of the individual branches, GFP—reporter gene.

An elementary classifier circuit providing a bell-shaped response in the two-dimensional input space can be constructed of two independent sensing branches, whose outputs are combined using a genetic AND gate (Fig 5) [16]. Each sensing branch is composed of two genetic modules, the sensor and the signal transducer [16]. The sensing module is monotonically induced by the corresponding input chemical signal *X*_*j*_ (*j* = 1,2) and drives the synthesis of an intermediate repressor/activator *U*_*j*_. The signal transducer part is activated by *U*_*j*_ at intermediate concentrations and inhibited at higher concentrations, providing the maximal response at a certain concentration level. The classic well-characterized example of such promoter is the promoter *P*_*RM*_ of phage lambda which provides this kind of non-monotonic response to the lambda repressor protein CI [26].

The outputs *Z*_*j*_ of both sensing branches drive the expression of a reporter protein (e.g., GFP) through a two-input genetic AND gate. A number of circuits performing logical operations including AND have been developed and characterized recently [22–24]. When each sensing branch provides a bell-shaped response function, then the response of the full circuit will also be a bell-shaped function in the two-dimensional input space.

Omitting the indices *j* at all variables and parameters for the sake of conciseness and denoting the concentrations of *X*, *U*, *Z* with *x*, *u* and *z*, the steady-state concentration of each single sensing branch output *Z* can be written as [16]

$$\begin{array}{c} z\left(x;m_{u},m_{z}\right)=\frac{r_{z}\left(r_{u}\left(x;m_{u}\right)/\mu_{u};m_{z}\right)}{\mu_{z}}, \end{array}$$ (11)

where *x* is the input concentration, *μ*_*u*_ and *μ*_*z*_ are the degradation rates of *U* and *Z*, respectively; *r*_*u*_(⋅) and *r*_*z*_(⋅) are the effective production rates of *U* and *Z* described by standard Hill functions

$$\begin{array}{c} r_{u}\left(x;m_{u}\right)=m_{u}\cdot \frac{\alpha A_{u}^{p_{u}}+x^{p_{u}}}{A_{u}^{p_{u}}+x^{p_{u}}}, \end{array}$$ (12)

$$\begin{array}{c} r_{z}\left(u;m_{z}\right)=m_{z}\cdot \frac{A_{z}^{p_{z}}u^{p_{z}}}{{\left(A_{z}^{p_{z}}+u^{p_{z}}\right)}^{2}}, \end{array}$$ (13)

where *α* determines the basal expression from the sensor promoter in the absence of the input chemical *X*, *A*_*u*_ and *A*_*z*_ are the dissociation constants of *X* and *U* with their corresponding promoters, the Hill coefficients *p*_*u*_ and *p*_*z*_ characterize the cooperativity of activation or repression of the corresponding promoters, *m*_*u*_ and *m*_*z*_ describe the overall expression strength of *U* and *Z*.

The function *z*(*x*) defined by Eqs (11)–(13) is bell-shaped in a range of *m*_*u*_/*μ*_*u*_ ∈ (*A*_*z*_,*A*_*z*_/*α*), with the position of the maximum determined by the value of *m*_*u*_/*μ*_*u*_[16]. A master population of elementary two-input classifiers with response maxima randomly varied in the input space can be constructed by random variation of the sensory promoter strengths *m*_*u*_ both among the individual cells, as well as among the two sensory branches in each cell. The variation range of the maximum position is limited by the parameter *α*, which is for common promoters of the order of 10^−3^[27, 28]. The full range can be covered, provided the promoter strengths *m*_*u*_ are varied at least 1/*α* = 10^3^ fold, which is achievable, for example, by varying the DNA sequence within and near the ribosome binding site of the sensory gene [17, 18].

In the following we let the *m*_*u*_ parameters of the two sensory branches in a chosen *i*th cell take on the values $m_{1}^{i}$ and $m_{2}^{i}$, the lower index denoting the input, the upper being the cell number, with all other parameters being the same in both sensory branches in all cells. We model the AND gate, which drives the reporter protein production, as a product of two Hill functions

$$\begin{array}{c} g\left(z_{1},z_{2}\right)=\beta \cdot \frac{z_{1}^{p_{g}}}{A_{g}^{p_{g}}+z_{1}^{p_{g}}}\cdot \frac{z_{2}^{p_{g}}}{A_{g}^{p_{g}}+z_{2}^{p_{g}}}, \end{array}$$ (14)

where *z*_1,2_ are the inputs to the AND gate, *β* is a dimensional constant, *A*_*g*_ and *p*_*g*_ are respectively the dissociation constant and the Hill coefficient for the AND gate (for simplicity we assume equal values for both inputs).

The inputs to the AND gate are essentially the outputs of the sensory branches, thus the output of a chosen *i*th cell finally is

$$\begin{array}{c} f_{i}\left(x_{1},x_{2}\right)=g\left(z\left(x_{1};m_{1}^{i}\right),z\left(x_{2};m_{2}^{i}\right)\right), \end{array}$$ (15)

where *x*_1,2_ are the classifier inputs, the function *g*(⋅, ⋅) is defined by Eq (14), and *z*(⋅) by Eqs (11)–(13) with *m*_*u*_ substituted by $m_{1}^{i}$ or $m_{2}^{i}$ for either input branch, and index *i* labeling the individual cells.

### Soft learning strategy

By “soft learning” we mean a learning strategy which reshapes the population density in the parameter space in response to a sequence of training examples in order to maximize the correct answer probability for the distributed classifier taken as a whole, without any hard separation of the cells into “correct” and “incorrect”.

This can be achieved by organizing a kind of population dynamics which gives preference to cells which tend to maximize the performance of the whole classifier. In the simplest case, the training examples are sequentially presented to all cells in the population, and some cells get eliminated from the population in a probabilistic way, with survival probability depending upon the cell output, given the *a priori* knowledge about the particular training example to belong to a certain class.

We use a more elaborate learning strategy incorporating a mechanism for conserving the total cell count. In the model description this is achieved by simply replacing each discarded cell with a duplicate of a randomly chosen cell from the population. In [16] it is shown that in the limit of infinite number of cells and infinite number of training samples the evolution of the population during this learning process is described by a set of ordinary differential equations in the form of a classical competition model. The viabilities of the competing cell types depend upon the correctness of their answers to the training samples. The same kind of dynamics can be approximately implemented in experiment, if the selection goes in parallel with cell division. The conserved number of cells is essentially the maximal (equilibrium) population size determined by experimental conditions. The cell viabilities can be controlled using FACS or by means of well-established genetically encoded positive/negative selection methods [29].

In consistency with [16], we specify the probabilities of cell survival after presenting each training example as

$$p_{+}\left(g\right)=\frac{1}{1+\xi }+\frac{1}{1+\xi \exp(-g/\gamma )},$$ (16a)

$$p_{-}\left(g\right)=\frac{1}{1+\xi }-\frac{1}{1+\xi \exp(-g/\gamma )}+1,$$ (16b)

where *g* is the cell output upon presenting a training example, *ξ* = exp(8*γ* ^−1^), *γ* controls the “softness” of the learning (the greater *γ*, the softer is the slope of *p*_+_(*g*) and *p*_−_(*g*)). Either *p*_+_(*g*) or *p*_−_(*g*) is used, depending on the class to which the training example is *a priori* known to belong. The functions specified in (Eqs 16a,16b) have maximal slope at *g* = 1/8. The cell output range should be scaled to cover this value by adjusting the constant *β* in Eq (14).

The output of a distributed classifier is the sum of all individual cell outputs:

$$\begin{array}{c} f\left(x_{1},x_{2}\right)=\sum_{i=1}^{N_{c}}f_{i}\left(x_{1},x_{2}\right), \end{array}$$ (17)

where *f*_*i*_(*x*_1_,*x*_2_) is defined by Eq (15), and *N*_*c*_ is the total number of cells.

The classification decision is made by comparing the classifier output to a threshold *θ*:

$$\begin{array}{c} \text{decision}=\{\begin{array}{cc} “\text{positive}”, & \text{if}\;f(x_{1},x_{2})\geq \theta ,\\ “\text{negative}”, & \text{if}\;f(x_{1},x_{2})<\theta , \end{array} \end{array}$$ (18)

where *θ* has to be adjusted after the learning to maximize the correct answer rate of the classifier.

The classification border is actually a level line of *f*(*x*_1_,*x*_2_) corresponding to the threshold *θ*. The aim of the soft learning is thus to reshape the population and select the optimal value of *θ* in a way that the corresponding level line is the best approximation of the (unknown *a priori*) optimal classification border. The computational criterion of this optimality is the maximization of the correct answer rate using the given training examples.

### Simulations

We used algorithm described in Table 2 to implement the soft learning strategy. We demonstrate the use of the soft classification strategy to solve two problems which are not solvable with hard distributed classifiers described in section “Hard classification problem”. The first example has separable classes which consist of disjoint regions and thus do not satisfy the requirements of convexity and negative slopes which were imposed in subsection “Hard classification technique and learning strategy”. The positive class is specified as union of two circles on the (*x*_1_,*x*_2_) plane, one centered at *x*_1_ = *x*_2_ = *A* with radius *R*, and the other centered at *x*_1_ = *x*_2_ = *B* with radius 3*R*, and the negative class as union of two ellipses, one centered at *x*_1_ = *A*, *x*_2_ = *B* with semiaxes *R* and 3*R*, and the other centered at *x*_1_ = *B*, *x*_2_ = *A* with semiaxes $3R\sqrt{2}$ and $R\sqrt{2}$, where

$$\begin{array}{c} R=\frac{1}{32}(1-10^{-1.5}),\;A=10^{-1.5}+2R,\;B=10^{-1.5}+8R. \end{array}$$ (19)

**Table 2:** Soft learning algorithm.

**Input:** Master population of *N*_*c*_ elementary classifiers (cells) with bell-shaped output with parameters $\left(m_{1}^{i},m_{2}^{i}\right)$ randomly sampled from the log-uniform distribution in the parameter space, bounded by the minimal and maximal values *m*_min_ and *m*_max_. The sequence of training examples $\left(x_{1}^{j},x_{2}^{j}\right)$ of length *N*_train_. The known class type *y* ^*j*^ = ±1 for each example. The number of training iterations *N*_iter_.
**Output:** Trained set of *N*_*c*_ cells constituting a distributed classifier; classification threshold *θ*_opt_.
**for** iteration *k* = 1 to *N*_iter_ **do**
Choose a random example $\left(x_{1}^{j},x_{2}^{j}\right)$.
**for** each cell *i* = 1 to *N*_*c*_ **do**
Calculate the *i*th cell output $g_{i}=f_{i}(x_{1}^{j},x_{2}^{j})$ according to Eq (15).
Calculate the cell survival probability according to Eq (16a) or Eq (16b): *p* = *p*_+_(*g*_*i*_) if *y*_*i*_ = +1, or *p* = *p*_−_(*g*_*i*_) if *y*_*i*_ = −1.
With probability 1−*p*, choose a random cell from the population and eliminate the *i*th cell, replacing it with the chosen cell.
**end for**
**end for**
**for** each training example *j* = 1 to *N*_train_ **do**
Use the trained population to calculate the population output $f(x_{1}^{j},x_{2}^{j})$ according to Eq (17).
**end for**
Find the optimal classification threshold *θ*_opt_ by maximizing the correct classification rate over *θ*: $\theta_{\text{opt}}=\text{argmax}\sum_{j=1}^{N_{\text{train}}}y^{j}\left[2H(f(x_{1}^{j},x_{2}^{j})-\theta )-1\right]$.

**Fig 6:**
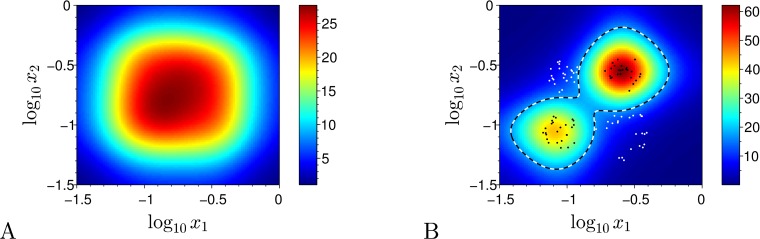
Simulation results for soft classification strategy applied to separable classes. (A) Untrained (master) population output (color). (B) Trained population output (color). White (black) filled circles—samples from the negative (positive) class, black-white dashed line—classification border of the trained classifier.

The simulation parameters are *N*_*c*_ = 2⋅10^3^, *N*_train_ = 100 (50 samples from each class), *N*_iter_ = 1000, softness parameter *γ* = 0.4, *m*_min_ = 2^2^ *A*_*z*_, *m*_max_ = 2^8^ *A*_*z*_, *A*_*z*_ = 20, *m*_*z*_ = *A*_*u*_ = 1, *A*_*g*_ = 2, *p*_*z*_ = *p*_*u*_ = *p*_*g*_ = 2, *α* = 10^−3^. Output scaling constant *β* = 1056.25 is chosen so that cell output *g* ranges from 0 to 0.25 in consistency with expressions for survival probabilities (16a,16b). The simulation result is presented in Fig 6. All training samples are classified correctly after learning, but this becomes impossible in case of inseparable classification problems.

**Fig 7:**
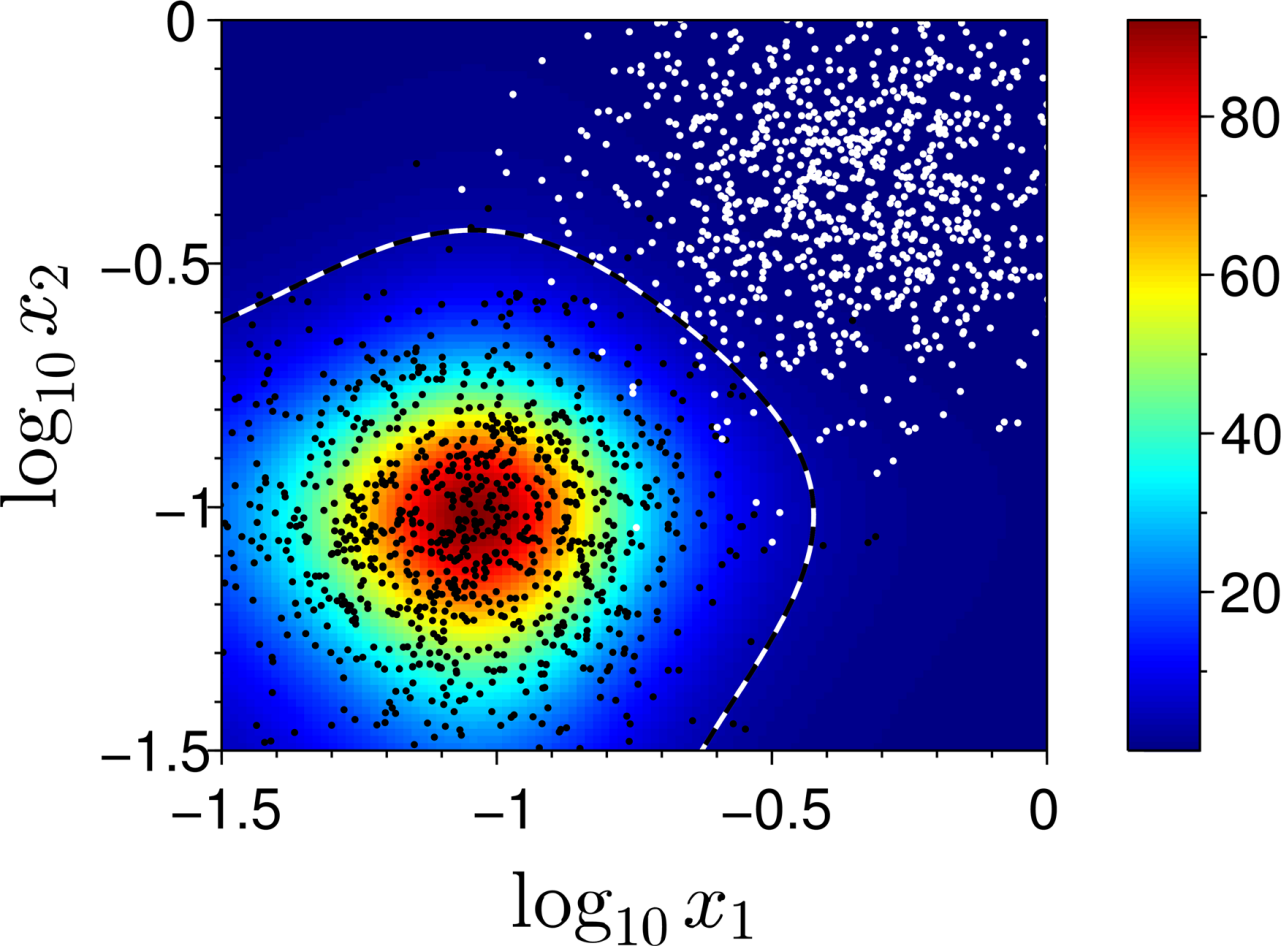
Simulation results for soft classification strategy applied to inseparable classes. Notations same as in Fig 6.

The next example shows the classifier operation for inseparable classes. For either class we use a two-dimensional log-normal distribution resulting from independently sampling both inputs *x*_1_ and *x*_2_ from a one-dimensional log-normal distribution centered at log*x*_1,2_ = −2.4 (log_10_ *x*_1,2_ ≈ −1.04) for the positive class, and at log*x*_1,2_ = −0.8 (log_10_ *x*_1,2_ ≈ −0.35) for the negative class, with standard deviation of log*x*_1,2_ for both classes set to 0.5 (which makes approximately 0.22 in terms of log_10_ *x*_1,2_). The length of the training sequence is *N*_train_ = 2000 (1000 samples from each class), chosen so that the distributions overlap is represented by a number of samples from both classes. Other simulation parameters are the same as in the previous example. The result of the simulation is presented in Fig 7 the same way as in the previous example. Since the training data are contradictory (overlapping), it is not possible to classify correctly all examples after learning. We observe, however, that the classification border produced by soft learning strategy separates the distributions close to a straight line equidistant from their centers in the logarithmic input space (which is the Bayesian solution).

The successful classification rate of the distributed classifier computed by validation against a testing sequence of length *N*_test_ = *N*_train_ = 2000 (1000 samples from each class) amounts to 98.35%, which is very close to the theoretical maximum of 98.82% set forth by the Bayesian classification rule. We performed another simulation with an increased overlap of the distributions, which is achieved by shifting the central point of the positive class to log*x*_1,2_ = −1.4 (log_10_ *x*_1,2_ ≈ −0.61), with all other conditions kept from the previous simulation. Validation against a test sequence yields performance 77.1%, while the Bayesian result is 80.19%.

There exist rigorous theorems on Bayes consistency (convergence to Bayesian decision boundaries) of classification methods [30], which applies to commonly used state-of-the-art machine learning methods. We did not carry out any rigorous consistency analysis for distributed classifiers, but based on the simulations we conclude that the considered mathematical model of distributed classifier demonstrates the possibility in principle to approach the theoretical maximum in classification performance. Biological implementation will face complications including cell count variation, transcriptional and instrumental noise, which inevitably cut down the classifier performance. That said, the simulation results justify the use of multi-input gene circuits in combination with distributed classifier approach to construct a learnable synthetic gene classifier.

## Discussion

In this paper we have presented a design of multi-input classifiers to be implemented as a synthetic genetic network. We have considered two examples, corresponding to hard and soft learning strategy. As a multi-input classifier, these devices can solve classification task based on the data inseparable in the single dimension case. Moreover, the proposed design allows to achieve practically arbitrary shape of the classification border in the space of input signals. Here we have considered two-input genetic classifiers but the same design principles can be utilized to construct multi-input classifying devices, then the number of inputs is limited only by the availability of orthogonal input-inducible promoters and multi-input hybrid promoters.

Our approach challenged a problem of discrimination between classes with overlapping probability density distributions in the input space. In this case the classification error probability cannot vanish and has to be minimized. The optimal solution to this problem is given by the Bayesian classification rule [31]. In case of equal *a priori* probabilities for a randomly picked sample to belong to either class, the classification of a presented sample point from the parameter space is optimally done by comparing the class probability density functions at this point: the class with the greatest probability density value is the optimal answer to the classification problem. At the classification border the probability density functions become equal. If these functions are known *a priori*, then the optimal border is thus also known, and the problem reduces to “hard classification”.

When the probability density functions of the classes are not known *a priori*, the optimal classification rule is not known either, and the classifier has to be trained by examples. Hard learning is not applicable in this case, because it may eventually lead to discarding all cells. Inseparable classes with *a priori* unknown probability density functions require another learning strategy which we refer to as “soft learning”, when the decision to discard or to keep a particular cell upon presenting a training example is probabilistic, depending on the cell output.

Our consideration did not account for cell division. An accurate description of population dynamics should incorporate the dependence of the cell division rate upon the metabolic burden imposed by the synthetic constructs. This factor can play a destructive role on classification, since non-uniform cell duplication may reshape the trained population in a way that distorts the classifier output. Judging whether this effect will be important for the experimental system requires quantitative understanding of metabolic burden imbalance and its impact upon cell division. We leave it for a separate study.

Another challenge to implementation is noise, both biological and instrumental. In [16] performance of distributed classifiers based on single-input genetic circuits was studied in the presence of both mentioned types of noise. It was shown that noise, when not too strong, does not destroy the classifier performance. We note, that a distributed classifier must be more robust to noise than single-cell classifiers due to averaging out the individual cell noise in the summary output. In this study we did not specially address the problem of noise. We do not expect, however, any qualitative difference in noise robustness between single- and multi-input classifier circuits.

An important aspect of synthetic biology is the design of smart biological devices or new intelligent drugs, through the development of in vivo digital circuits [32]. If living cells can be made to function as computers, one could envisage, for instance, the development of fully programmable microbial robots that are able to communicate with each other, with their environment and with human operators. These devices could then be used, e.g., for detection of hazardous substances or even to direct the growth of new tissue. In that direction, pioneering experimental studies have shown the feasibility of programmed pattern formation [9], the possibility of implementing logical gates and simple devices within cells [33], and the construction of new biological devices capable to solve or compute certain problems [34].

The classifiers designed could be considered as a further development towards the construction of robust and predictable synthetic genetic biosensors, which have the potential to affect and effect a lot of applications in the biomedical, therapeutic, diagnostic, bioremediation, energy-generation and industrial fields [35–38].

## Supporting Information

S1 Appendix Deriving an estimate for hard classifier response. (PDF)
