# Supplementary material for: Multi-Input Distributed Classifiers for Synthetic Genetic Circuits
Source: PLoS One. 2015 May 6;10(5):e0125144. doi: 10.1371/journal.pone.0125144 (PMC4450813; doi:10.1371/journal.pone.0125144)
Supplement: S1 Appendix — (PDF) [file pone.0125144.s001.pdf]

Appendix S1. Deriving an estimate for hard classifier response

We assume that the master population is characterized by a "working parameter domain" in the space of parameters (m_1, m_2), such that the density of cells per logarithmic unit of the parameter space in this working domain is greater than or equal to a known minimal value α. Precisely, we assume that the expected number of cells dN falling within a parameter space element $dm_1 dm_2$ satisfies the following inequality everywhere in the working parameter domain:

$$dN \geq \alpha \cdot d(\log m_1)\, d(\log m_2). \tag{S1.1}$$

Below we derive a lower estimate for $N_{pos}(a_1^{\text{in}}, a_2^{\text{in}})$ which is the expectation of the number of cells answering positively to an input $(a_1^{\text{in}}, a_2^{\text{in}})$ taken from the positive class:

$$N_{pos}(a_1^{\text{in}}, a_2^{\text{in}}) > \frac{2\alpha\delta^2}{(1+\delta)^2}, \tag{S1.2}$$

where δ is a parameter determining the offset of the input from the classification border into the positive class. In general, δ is defined in a way that $(1+\delta)$ is a factor by which the negative region has to be scaled so as its border reaches the input point (see details below). Using polar coordinates (ρ, ϕ) defined by $a_1 = \rho\cos\phi$, $a_2 = \rho\sin\phi$, we can define δ in the form

$$\delta = \frac{\rho^{\text{in}}}{\rho_b(\phi^{\text{in}})} - 1, \tag{S1.3}$$

where $\rho = \rho_b(\phi)$ is the border equation, and $(\rho^{\text{in}}, \phi^{\text{in}})$ is the input point:

$$\begin{aligned} a_1^{\text{in}} &= \rho^{\text{in}}\cos\phi^{\text{in}}, \\ a_2^{\text{in}} &= \rho^{\text{in}}\sin\phi^{\text{in}}. \end{aligned} \tag{S1.4}$$

The derivation of (S1.2) is based upon the assumption that the whole region of the parameter space associated with producing the positive output of the trained classifier is contained in the working parameter domain, and hence is populated by cells with density satisfying (S1.1). This is actually the only requirement limiting the applicability of (S1.2).

Below we analyze this requirement in case of the working parameter domain specified as a rectangle $\{m_{\min} \leq m_{1,2} \leq m_{\max}\}$. We derive a set of conditions providing the applicability of (S1.2) to a particular input $(a_1^{\text{in}}, a_2^{\text{in}})$:

$$\mu_1^* \leq m_{\max}, \quad \mu_2^* \leq m_{\max}, \tag{S1.5a}$$

$$m_{\min} a_1^{\text{in}} + \mu_2^* a_2^{\text{in}} \leq 1, \tag{S1.5b}$$

$$\mu_1^* a_1^{\text{in}} + m_{\min} a_2^{\text{in}} \leq 1. \tag{S1.5c}$$

with μ_1^* and μ_2^* defined as the coefficients of an equation in the form

$$\mu_1^* a_1 + \mu_2^* a_2 = 1, \tag{S1.6}$$

describing the tangent to the border drawn at the point where it is crossed by the input's radius vector.

Conditions (S1.5a-c) are given a geometrical interpretation and can be used for choosing parameter values in experiment and simulations. Condition (S1.5a) can be formulated in terms of the border intercepts (the abscissa and the ordinate of the points where the border crosses the axes). Namely, $m_{\max}$ should not be less than the inverse of each intercept. The interpretation of (S1.5b,c) is less straightforward, but it suggests that these conditions fail whenever the input point is too close to either axis, or the tangent

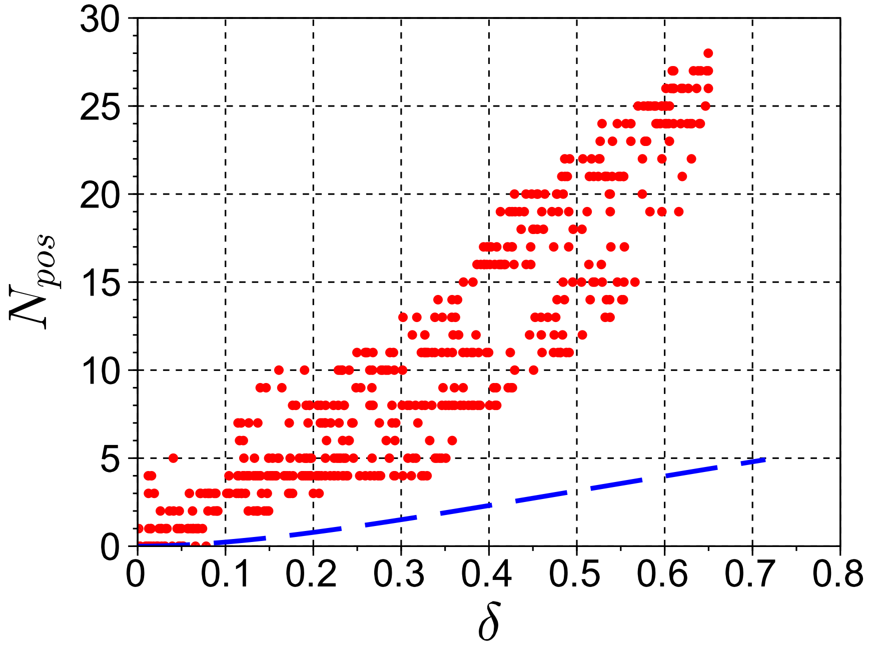

Figure S1. Validating the estimate for hard classifier response. Number of positively responding cells N_{pos} versus the input offset δ from the class border. Red circles – simulation results, blue dashed line – lower estimate (S1.2).

to the border drawn at the point where it is crossed by the input radius vector is too close to being parallel to either axis. At the same time, (S1.5b,c) are the less likely to fail, the smaller is $m_{\min}$, and the closer is the input to the border (which implies smaller δ).

We stress that the lower estimate in (S1.2) is obtained for expectations, and the actual count of the positively answering cells is a random variate determined by a particular realization (scattering) of the master population in the parameter space.

To validate the estimate (S1.2), we extended the simulation described in section "Hard classification problem" by testing the trained classifier against a sequence of $N_{\text{test}} = 500$ samples from the positive class (black filled circles in Fig. 4 in main text). For each input $(a_1^{\text{in}}, a_2^{\text{in}})$ we calculate the corresponding δ according to (S1.3), (S1.4), and measure the quantity of the positively responding cells $N_{\text{pos}}(a_1^{\text{in}}, a_2^{\text{in}})$. The obtained set of pairs N_{pos} versus δ is plotted with red filled circles in Fig. S1. The analytical lower estimate (S1.2) is plotted with a blue dashed line.

In order to derive (S1.2), we first notice that each particular input $(\tilde{a}_1, \tilde{a}_2)$ can be associated with a straight line on the parameter plane (m_1, m_2), defined by the equation

$$m_1 \tilde{a}_1 + m_2 \tilde{a}_2 = 1. \tag{S1.7}$$

Consider a polygonal classification border (satisfying the requirements of negative slopes and convexity) and an input (a_1^0, a_2^0) lying exactly on the border, namely, on its ith segment, and satisfying the ith segment's equation:

$$\mu_1^i a_1^0 + \mu_2^i a_2^0 = 1. \tag{S1.8}$$

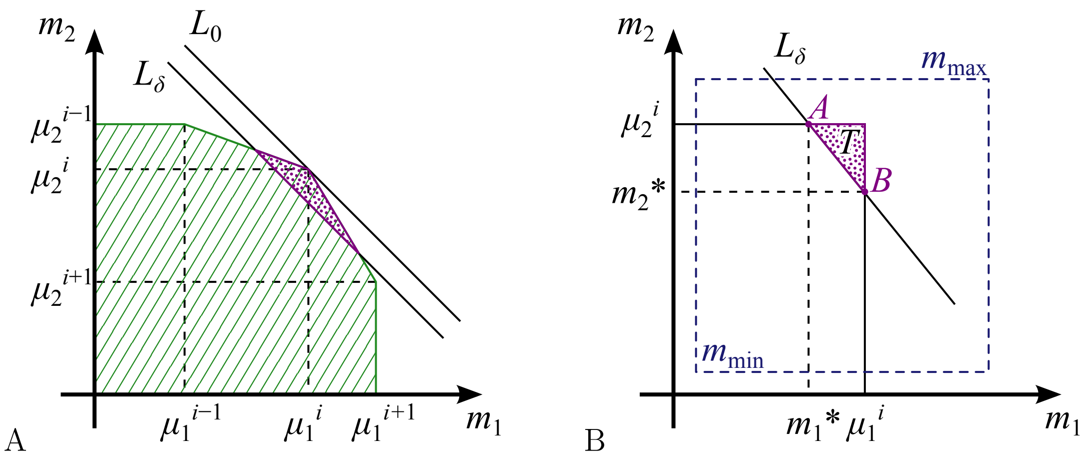

Figure S2. Estimating the number of positively responding cells. (A) Hatched area – trained ensemble region, dotted area – cells answering positively to an input sample from the positive class. (B) Dashed square – working parameter domain. Other notations – see text.

The corresponding line L_0 defined by (S1.7) with $\tilde{a}_1 = a_1^0$, $\tilde{a}_2 = a_2^0$ then touches the trained ensemble region on the parameter plane at the vertex (μ_1^i, μ_2^i) (Fig. S2A).

Now consider another input $(a_1^{\text{in}}, a_2^{\text{in}})$, which is slightly shifted from the border into the positive class, namely, satisfying

$$\mu_1^i a_1^{\text{in}} + \mu_2^i a_2^{\text{in}} = 1 + \delta, \tag{S1.9}$$

where $\delta > 0$ is a parameter determining the offset of the input point from the negative class. The line L_δ defined by (S1.7) with $\tilde{a}_1 = a_1^{\text{in}}$, $\tilde{a}_2 = a_2^{\text{in}}$ then crosses the trained ensemble region (Fig. S2A).

Let us estimate the expectation $N_{pos}(a_1^{\text{in}}, a_2^{\text{in}})$ of the number of cells in the trained ensemble which answer positively to the input $(a_1^{\text{in}}, a_2^{\text{in}})$. We note that the individual cells answering positively to this input are exactly those, whose parameters are located in the trained ensemble region above the line L_δ (dotted area in Fig. S2A).

We also notice that the rectangle $\{0 < m_1 \le \mu_1^i, 0 < m_2 \le \mu_2^i\}$ is always a subset of the trained ensemble region (see Fig. S2A). Denote with T a piece of this rectangle which is cut from it by the line L_δ (dotted area in Fig. S2B):

$$T = \left\{m_1, m_2 : 0 < m_1 \le \mu_1^i, 0 < m_2 \le \mu_2^i, m_1 a_1^{\text{in}} + m_2 a_2^{\text{in}} > 1\right\}. \tag{S1.10}$$

Denoting the expectation of the cell count in T with $N_T(a_1^{\text{in}}, a_2^{\text{in}})$, we observe that it is a lower estimate for $N_{pos}(a_1^{\text{in}}, a_2^{\text{in}})$:

$$N_{pos}(a_1^{\text{in}}, a_2^{\text{in}}) \ge N_T(a_1^{\text{in}}, a_2^{\text{in}}). \tag{S1.11}$$

We consider the case when T is a triangle (not a trapezium) and justify this assumption below.

We express $N_T(a_1^{\text{in}}, a_2^{\text{in}})$ as an integral

$$N_T(a_1^{\text{in}}, a_2^{\text{in}}) = \int_T f(m_1, m_2)\, dm_1 dm_2, \tag{S1.12}$$

where $f(m_1, m_2)$ is the "cell density function" in the parameter space (m_1, m_2). Since the minimal cell density α in the logarithmic parameter space is specified by (S1.1), the cell density function satisfies

$$f(m_1, m_2) \ge \frac{\alpha}{m_1 m_2}, \tag{S1.13}$$

as soon as the pair (m_1, m_2) belongs to the working parameter domain. We assume that (S1.13) holds in the whole area of T and provide a sufficient condition for this below.

As $f(m_1, m_2)$ in (S1.13) is falling in both arguments, the integral in (S1.12) can be given a lower estimate

$$N_T(a_1^{\text{in}}, a_2^{\text{in}}) > f(\mu_1^i, \mu_2^i) \cdot S(T) \geq \frac{\alpha\delta^2}{2\mu_1^i\mu_2^i a_1^{\text{in}} a_2^{\text{in}}}, \tag{S1.14}$$

where $S(T) = \delta^2/(2a_1^{\text{in}}a_2^{\text{in}})$ is the area of the triangle T. Taking into account the inequality of arithmetic and geometric means, which along with (S1.9) yields

$$\mu_1^i\mu_2^i a_1^{\text{in}} a_2^{\text{in}} \leq (\mu_1^i a_1^{\text{in}} + \mu_2^i a_2^{\text{in}})^2/4 = (1+\delta)^2/4,$$

and combining (S1.14) with (S1.11), we finally arrive at (S1.2).

The input offset parameter δ is introduced in (S1.9). Geometrically, the input point $(a_1^{\text{in}}, a_2^{\text{in}})$ is located on a straight line which results from scaling the line drawn through the ith border segment (defined by (S1.8)) by a factor of $(1+\delta)$ with the transform center placed at the origin of the coordinates. This could be used as a definition for δ, but the choice of the "ith segment" itself may be ambiguous. However, the calculation remains valid regardless of this particular choice. To obtain the best (highest) estimation in (S1.2), we should choose the segment number i which maximizes δ in (S1.9) for a given input.

To solve this maximization problem, consider a uniform scaling of the negative region (with the transform center at the coordinates origin) by a factor of s, such that the input point $(a_1^{\text{in}}, a_2^{\text{in}})$ becomes located on the scaled class border (the negative class is "inflated" till it reaches the input point). Denote with l the number of the border segment, which hits the input point when the border is scaled. It means that the following equation is satisfied:

$$\frac{\mu_1^l}{s} a_1^{\text{in}} + \frac{\mu_2^l}{s} a_2^{\text{in}} = 1. \tag{S1.15}$$

At the same time, due to the convexity of the negative region, for all segments of the scaled border the following inequality holds:

$$\frac{\mu_1^i}{s} a_1^{\text{in}} + \frac{\mu_2^i}{s} a_2^{\text{in}} \leq 1 \tag{S1.16}$$

with the equality taking place only for $i = l$ and, in the special case when the input hits a vertex of the scaled polygon, for two adjacent segments. Comparing (S1.15) and (S1.16) to (S1.9), we conclude that δ in (S1.9) is maximized at $i = l$, and this maximal value satisfies $1 + \delta = s$. Essentially, the "optimal" segment $i = l$ is the one which is crossed by the input's radius vector (or just a straight line segment drawn from the coordinates origin to the input point).

Thus, for an arbitrary given polygonal classification border (satisfying the requirements of convexity and negative slopes) the best estimate in (S1.2) is obtained, when δ is defined in a way that $(1+\delta)$ is a factor by which the negative region has to be "inflated" (i.e. scaled up in the transformed input space (a_1, a_2) with the origin of the coordinates used as the scaling transform center), so as the input point finds itself on the scaled classification border. This definition of δ remains equally valid in the limit of a smooth border. Using polar coordinates, we can express this definition in the form (S1.3).

In the derivation of (S1.2) exactly two assumptions were made: (i) T being a triangle, and (ii) cell density estimation (S1.13) valid in the whole area of T. Let us check their applicability for a given input $(a_1^{\text{in}}, a_2^{\text{in}})$.

In case of a polygonal border, denote with $\mu_{1,2}^* = \mu_{1,2}^l$ the equation coefficients of the "optimal" border segment $i = l$ (crossed by the input's radius vector), identified in (S1.15). In the smooth border limit, instead of the optimal border segment one can speak of the "optimal" border tangent, drawn at the point where the border is crossed by the input's radius vector. In this case we denote with $\mu_{1,2}^*$ the equation coefficients of this optimal tangent.

Denote with m_1^* the value of m_1 at the crossing point of the lines L_δ and $m_2 = \mu_2^*$ (i.e., abscissa of the point A in Fig. S2B), and with m_2^* the value of m_2 at the crossing point of the lines L_δ and $m_1 = \mu_1^*$ (i.e., ordinate of B in Fig. S2B):

$$m_1^* = \frac{1 - \mu_2^* a_2^{\text{in}}}{a_1^{\text{in}}}, \quad m_2^* = \frac{1 - \mu_1^* a_1^{\text{in}}}{a_2^{\text{in}}}. \tag{S1.17}$$

Assume that the working parameter domain (where (S1.13) holds) is specified as a rectangle $\{m_{\min} \le m_{1,2} \le m_{\max}\}$. Using the above notations, we write down the conditions

$$\mu_1^* \le m_{\max}, \quad \mu_2^* \le m_{\max}, \tag{S1.18}$$
$$m_1^* \ge m_{\min}, \quad m_2^* \ge m_{\min}, \tag{S1.19}$$

which provide that T is a subset of the working parameter domain, and a corollary fact is T being a triangle (since $m_{1,2}^* > 0$ automatically). Inserting (S1.17) into (S1.19), we rewrite it in the form

$$\begin{aligned} m_{\min} a_1^{\text{in}} + \mu_2^* a_2^{\text{in}} &\le 1, \\ \mu_1^* a_1^{\text{in}} + m_{\min} a_2^{\text{in}} &\le 1. \end{aligned} \tag{S1.20}$$

Combining (S1.18) with (S1.20) yields the set of conditions (S1.5a-c).

These conditions can be given a geometrical interpretation. Rewriting the equation of a border tangent in the intercept form instead of the form (S1.6) yields the intercepts of the tangent (the abscissa and the ordinate of the points where it crosses the axes), which are $1/\mu_1^*$ and $1/\mu_2^*$. The minimal values of these intercepts are actually the intercepts of the border itself (due to convexity). Therefore, the maximal values of μ_1^* and μ_2^* can be found as the inverse of the border intercepts. Condition (S1.18) then reduces to the requirement that m_{max} should not be less than the inverse of each intercept.

The interpretation of (S1.20) is less straightforward. Consider the point where the class border is crossed by the input's radius vector, and the border tangent drawn at this point, earlier referred to as the "optimal tangent", whose equation coefficients are μ_1^* and μ_2^*. Denote with d the length of the tangent segment belonging to the first quadrant and clipped by the axes. This segment is split into two sections by the tangency point. Denote with d_1 and d_2 the lengths of these sections adjacent to the axes Oa_1 and Oa_2, respectively, so that $d = d_1 + d_2$. Then a geometrical calculation yields

$$\mu_1^* a_1^{\text{in}} = (1+\delta)\frac{d_2}{d}, \quad \mu_2^* a_2^{\text{in}} = (1+\delta)\frac{d_1}{d}. \tag{S1.21}$$

Inserting (S1.21) into (S1.20), we obtain

$$\begin{aligned} m_{\min} a_1^{\text{in}} + \delta &\le \frac{d_2}{d}(1+\delta), \\ m_{\min} a_2^{\text{in}} + \delta &\le \frac{d_1}{d}(1+\delta). \end{aligned} \tag{S1.22}$$

This condition favors smaller δ and $m_{\min}$, but fails whenever d_1/d or d_2/d becomes too small. This is the case, when the input is close to either axis, or the tangent to the border drawn at the point where it is crossed by the input radius vector is too close to being parallel to either axis.
